# Unusual tertiary pairs in eukaryotic tRNA^Ala^

**DOI:** 10.1261/rna.076299.120

**Published:** 2020-11

**Authors:** Eric Westhof, Shubo Liang, Xiaoling Tong, Xin Ding, Lu Zheng, Fangyin Dai

**Affiliations:** 1 State Key Laboratory of Silkworm Genome Biology, College of Biotechnology, Southwest University, Chongqing 400715, China; 2 Architecture et Réactivité de l'ARN, Institut e Biologie Moléculaire et Cellulaire, UPR9002 CNRS, Université de Strasbourg, Strasbourg 67084, France

**Keywords:** tRNA, anticodons, Ala, Gly, insects, mammals

## Abstract

tRNA molecules have well-defined sequence conservations that reflect the conserved tertiary pairs maintaining their architecture and functions during the translation processes. An analysis of aligned tRNA sequences present in the GtRNAdb database (the Lowe Laboratory, University of California, Santa Cruz) led to surprising conservations on some cytosolic tRNAs specific for alanine compared to other tRNA species, including tRNAs specific for glycine. First, besides the well-known G3oU70 base pair in the amino acid stem, there is the frequent occurrence of a second wobble pair at G30oU40, a pair generally observed as a Watson–Crick pair throughout phylogeny. Second, the tertiary pair R15/Y48 occurs as a purine–purine R15/A48 pair. Finally, the conserved T54/A58 pair maintaining the fold of the T-loop is observed as a purine–purine A54/A58 pair. The R15/A48 and A54/A58 pairs always occur together. The G30oU40 pair occurs alone or together with these other two pairs. The pairing variations are observed to a variable extent depending on phylogeny. Among eukaryotes, insects display all variations simultaneously, whereas mammals present either the G30oU40 pair or both R15/A48 and A54/A58. tRNAs with the anticodon 34A(I)GC36 are the most prone to display all those pair variations in mammals and insects. tRNAs with anticodon Y34GC36 have preferentially G30oU40 only. These unusual pairs are not observed in bacterial, nor archaeal, tRNAs, probably because of the avoidance of A34-containing anticodons in four-codon boxes. Among eukaryotes, these unusual pairing features were not observed in fungi and nematodes. These unusual structural features may affect, besides aminoacylation, transcription rates (e.g., 54/58) or ribosomal translocation (30/40).

## INTRODUCTION

Transfer RNA specific for alanine has a long history. Fifty-five years ago, the sequence of yeast tRNA^Ala^ was published by Holley et al. (1965). The following year, the sequence of yeast tRNA^Tyr^ led to the establishment of the cloverleaf secondary structure of transfer RNAs (Madison et al. 1966). Since the early days of the structure determination of tRNA molecules (Quigley and Rich 1976; Hingerty et al. 1978; Sussman et al. 1978), the structural roles of tertiary base pairs for the maintenance of the L-shape fold characteristic of tRNAs are well-appreciated. Further sequence and structure analyses led to additional conservation within the anticodon loop (Yarus 1982; Auffinger and Westhof 2001). Although we do not have a structure prototype for each of the basic 20 native tRNAs in any system (and by far), the sequence conservations overwhelmingly support the occurrence of most (if not all) identified tertiary pairs in all tRNA species (with variations for the long-arm tRNAs) (Biou et al. 1994; Westhof and Auffinger 2012). It has been remarked that tRNAs specific for a given amino acid are very similar throughout phylogeny, whereas, in contrast, within a given organism, the various tRNA species differ more between each other (Goodenbour and Pan 2006; Saks and Conery 2007; Ardell 2010). Clearly, tRNAs within a cell need to be distinct from each other in order to prevent misacylation by noncognate aminoacyl tRNA synthetases, and synthetases are known to possess characteristic determinants for specifically recognizing a single tRNA species (Saks et al. 1994; Giege et al. 1998). In other words, a tRNA sequence has stronger linkages to the amino acid it charges than to the organism in which it is encoded. Simultaneously, tRNAs need to maintain nucleotide conservations for folding, recognition by elongation factors like EF-Tu (Schrader and Uhlenbeck 2011), and, in eukaryotes, defined sequences are conserved in the internal promoters (A and B boxes) for transcription by polymerase III (Marck et al. 2006; Mitra et al. 2015). While searching and analyzing through the sequence alignments presented in the genomic tRNA database (version August 2019) (Chan and Lowe 2016), it came therefore as a surprise that some cytosolic tRNA^Ala^ presented different nucleotide conservations throughout phylogeny.

## RESULTS

### tRNA sequence alignments

**FIGURE 1.**
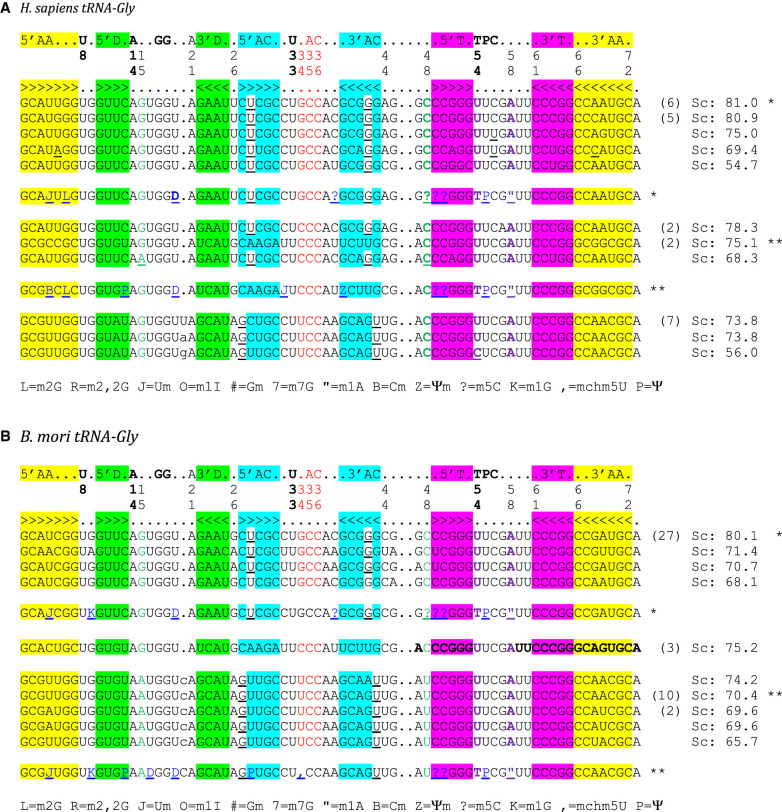

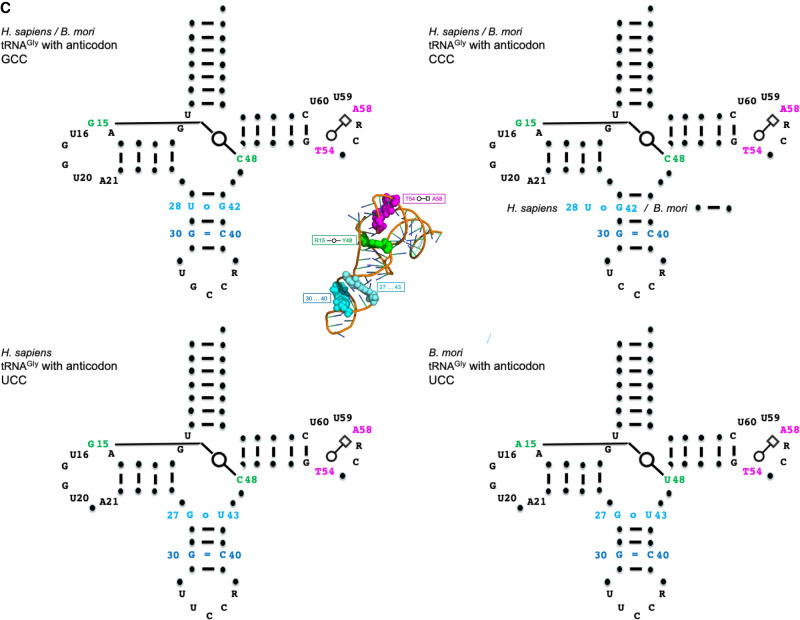
Structural alignments of tRNA^Gly^ from *Homo sapiens* (*A*) and *Bombyx mori* (*B*). The secondary structural elements of the tRNA cloverleaf are indicated *above* the alignments with key positions numbered following the standard nomenclature of yeast tRNA^Phe^. For clarity, the base-paired stems are colored (yellow for the amino acid stem; green for the dihydrouridine stem; cyan for the anticodon stem; purple for the thymine stem throughout the alignments), except for GoU pairs in the anticodon stem, which are white and underlined. The anticodon triplet is shown in red. At the *right*, the scores (Sc) from the tRNAscan-SE prediction algorithm (Lowe and Eddy 1997) as indicated in the database of transfer RNA genes GtRNAdb 2.0 (Chan and Lowe 2016) are shown. The number of potential tRNA gene copies is indicated next to the scores when greater than 1. Thus, for tRNA^Gly^, there are 14 isoacceptors with anticodon GCC, five with CCC anticodons, and nine with UCC anticodons. The two tertiary pairs extensively discussed in the text are in bold and colored—green for 15/48 and magenta for 54/58. The same color code is used in the 2D and 3D representations. The sequences of the two isoacceptor tRNA^Gly^ present in the MODOMICS database (Boccaletto et al. 2018) are shown, and the code used is explained in the last line. D stands for dihydrouridine and I for inosine. The starred sequences are, respectively, the predicted and the experimentally determined sequences for a given isoacceptor. (*C*) Standard cloverleaf structures corresponding to the alignments with key contacts highlighted with the same color code. The Leontis–Westhof (2001) nomenclature is used for the non-Watson–Crick pairs. In the *middle*, a representative three-dimensional structure of a tRNA (PDB 1EHZ from Shi and Moore 2000) is shown together with base pairs discussed.

We focus our analysis here on mammals and insects especially for tRNA^Gly^ and tRNA^Ala^, because for these two tRNAs the codon:anticodon triplets are G/C-rich and both belong to four-codon boxes. Besides, these two amino acids are the main components of silk-like fibers produced by several insects. Figure 1A,B shows the tRNA alignments, respectively, for *Homo sapiens* and *Bombyx mori* tRNA^Gly^. In the text, we use the symbols “-” for A-U/U-A pair, “=” for G = C/C = G, “o” for wobble, and “/” for non-Watson–Crick pairs. The stems are colored, and the highly conserved residues are in bold. The numbering follows the usual tRNA-Phe nomenclature. Thus, the residues U8, A14, G18 and G19, U33, U54 (modified generally in T or thymine), U55 (modified in Ψ or pseudouridine), and C56 stand out. The residues in lowercase are weakly conserved or not at all in the Infernal covariance model (Nawrocki and Eddy 2013). It can be observed also that at the lower end of the scores, unexpected nucleotides appear (underlined) in those highly conserved positions. It is interesting to note the subtle variations in the base pairings and conservations as a function of the anticodon. For example, with the anticodon GCC, there is a U28oG42 pair (and C38 in the anticodon loop), whereas with the anticodon UCC, there is a G27oU43 (and A38 in the anticodon loop) in both *H. sapiens* and *B. mori*. Notice also the change from G15/C48 to A15/U48 in *B. mori* UCC-tRNAGly. The corresponding cloverleaf representations of the main nucleotide conservations are shown in Figure 1C, color-coded as in Figure 1A,B. Besides the presence of some GoU pairs within the AC-stem, all other features of tRNA^Gly^ follow the known nucleotide conservations, in marked contrast to what is observed for tRNA^Ala^.

**FIGURE 2.**
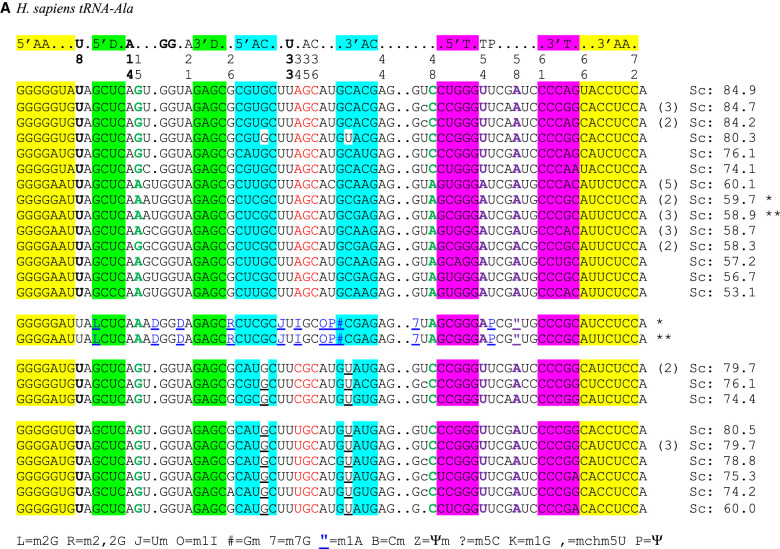

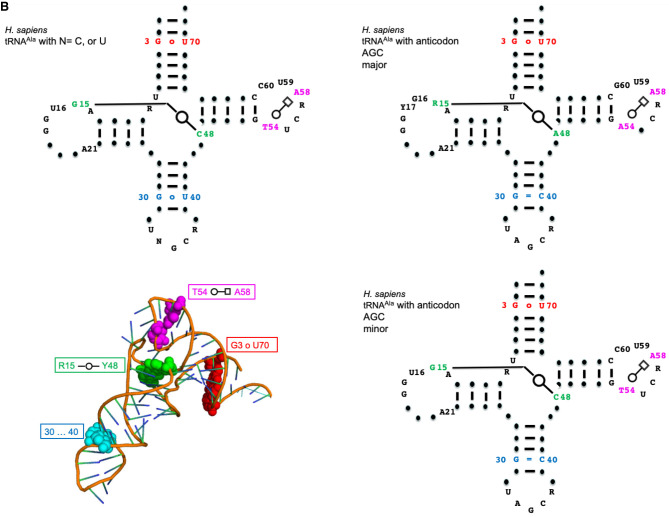

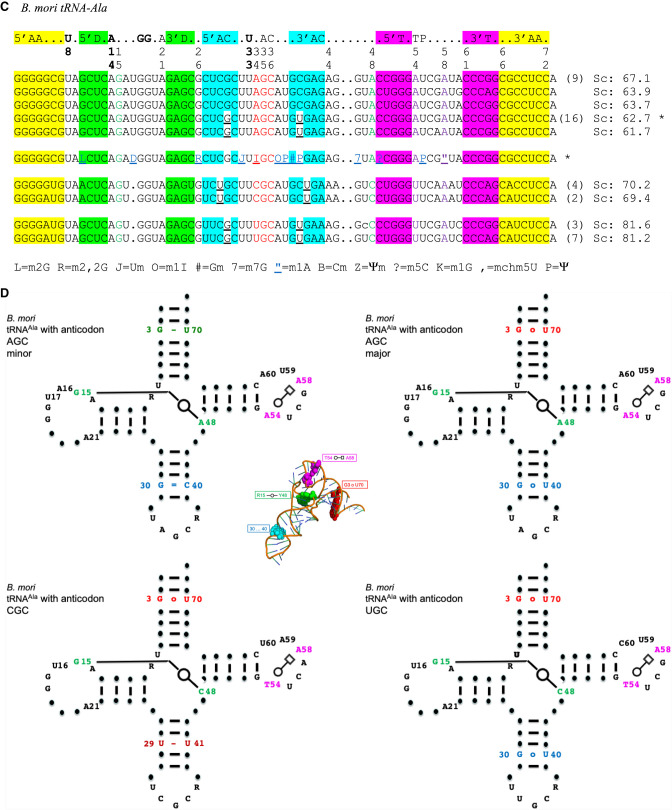
Structural alignments of tRNA^Ala^ from *Homo sapiens* (*A*) and *Bombyx mori* (*C*). The secondary structural elements of the tRNA cloverleaf are indicated *above* the alignments with key positions numbered following the standard nomenclature of yeast tRNA^Phe^. For clarity, the base-paired stems are colored (yellow for the amino acid stem; green for the dihydrouridine stem; cyan for the anticodon stem; purple for the thymine stem throughout the alignments), except for GoU pairs in the anticodon stem, which are white and underlined. The anticodon triplet is shown in red. At the *right*, the scores (Sc) from the tRNAscan-SE prediction algorithm (Lowe and Eddy 1997) as indicated in the database of transfer RNA genes GtRNAdb 2.0 (Chan and Lowe 2016) are shown. The number of potential tRNA gene copies is indicated next to the scores when greater than 1. The two tertiary pairs extensively discussed in the text are bold and colored—green for 15/48 and magenta for 54/58. The same color code is used in the 2D and 3D representations. The sequences of the two isoacceptor tRNA^Ala^ present in the MODOMICS database (Boccaletto et al. 2018) are shown, and the code used is explained in the last line. D stands for dihydrouridine and I for inosine. The starred sequences are, respectively, the predicted and the experimentally determined sequences for a given isoacceptor. (*B*,*D*) Standard cloverleaf structures corresponding to the alignments with key contacts highlighted with the same color code. The Leontis–Westhof (2001) nomenclature is used for the non-Watson–Crick pairs. A representative three-dimensional structure of a tRNA (PDB 1EHZ from Shi and Moore 2000) is shown together with base pairs discussed in each case.

In tRNA^Ala^, the key determinant for its cognate aminoacyl tRNA synthetase is a G3oU70 wobble pair in the amino acid stem (Hou and Schimmel 1988; McClain et al. 1988; Naganuma et al. 2014) and not the anticodon triplet (Giégé et al. 1998). This feature led to extensive studies mainly on the *Escherichia coli* system (Hou and Schimmel 1988, 1989; Hou et al. 1995). The base pair between residues 15 and 48 was especially studied (Hou et al. 1993, 1995). Nowadays, with so many more genomes available remarkably annotated (Chan and Lowe 2016), the analysis can be deepened. Figure 2A,C presents the alignments corresponding to tRNA^Ala^ in *H. sapiens* and *B. mori*. The tRNA sequences with the modifications of the two sets of tRNAs discussed (Ala and Gly) as extracted from MODOMICS (Boccaletto et al. 2018) are indicated in the figures. The corresponding cloverleaf representations of the main nucleotide conservations are shown in Figure 2B,D color-coded as in Figure 2A,C. We will now describe these new features.

### Unusual structural features in eukaryotic tRNA^Ala^

#### Two pairs in the secondary structure are unusual

##### The G3oU70 wobble pair in the amino acid stem

The first conserved unusual and well-documented feature in tRNA^Ala^ is the G3oU70 base pair as discussed above (Hou and Schimmel 1988, 1989; McClain et al. 1988; Hou et al. 1995; Naganuma et al. 2014). We did observe it in all alignments studied and we will not discuss it here further.

##### The G30oU40 wobble pair in the anticodon stem

The second unusual secondary pair is the presence of a GoU pair in the anticodon stem between nucleotides 30 and 40 in eukaryotic tRNA^Ala^. This base pair is generally mainly a G = C pair, or, less frequently, a C = G pair, in the vast majority of tRNAs across phylogeny (Grosjean and Westhof 2016). In humans or insects, several tRNA^Ala^ isodecoders present a G30oU40 base pair. In *B. mori*, a U29oU41 pair occurs in tRNAs with anticodon CGC. Surprisingly, the only occurrence of a wobble pair at 30–40 occurs in eukaryotic tRNA^Ile^ but in the reverse order: between U30 and G40. Interestingly, in the same codon box, tRNA^Met^ presents a U31–U39 in eukaryotes.

##### Residues 30 and 40 make contacts with the ribosome in the P-state

**FIGURE 3.**
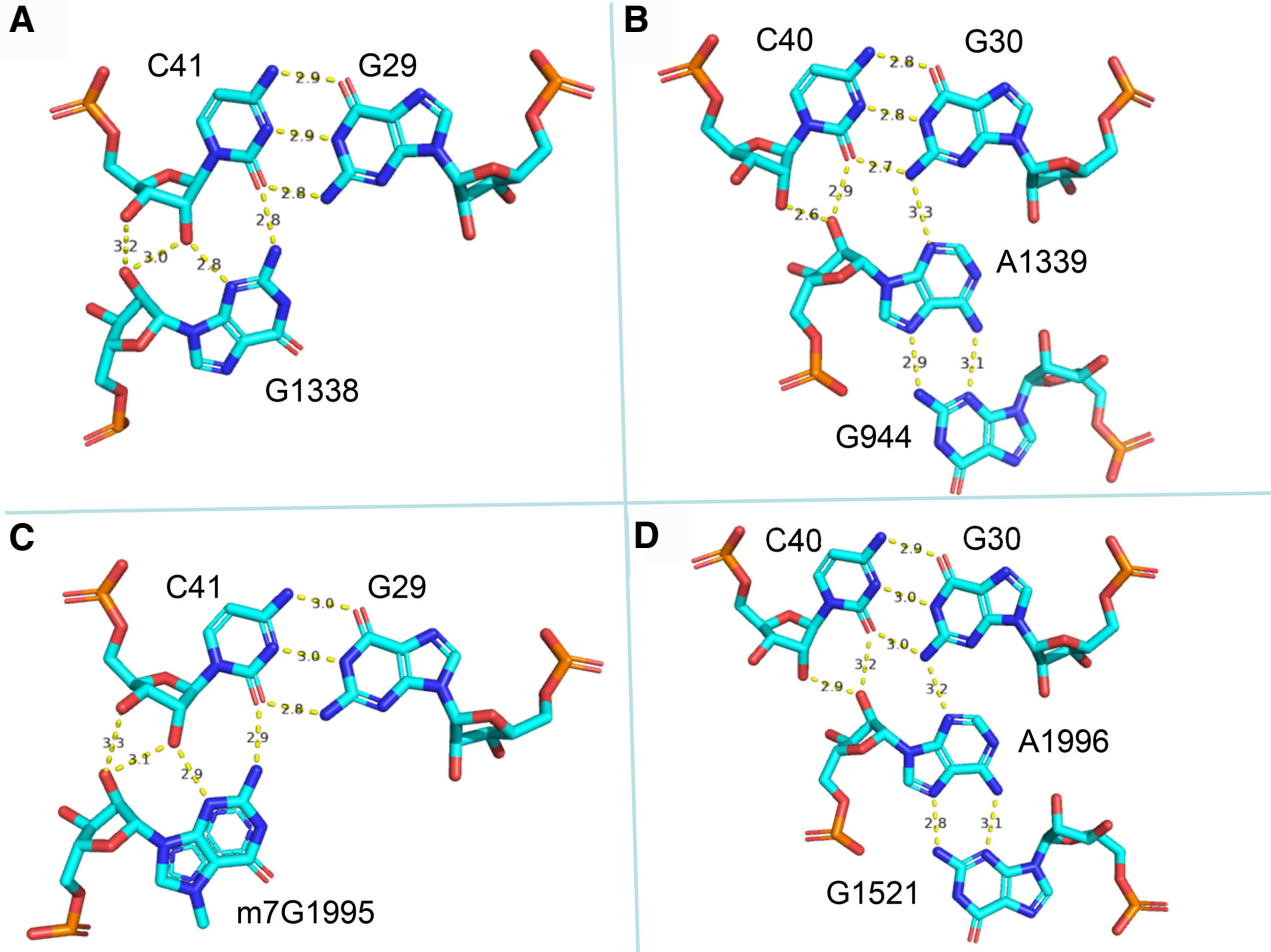
Interactions between the P-site tRNA and the large rRNA in bacterial ribosome (*A*,*B*) (from Watson et al. 2020) and in a eukaryotic ribosome (*C*,*D*) (from PDB 6AZ1, 6AZ3; Shalev-Benami et al. 2017). The contacts occur in the minor groove of base pairs 29–41 and 30–40 in the anticodon stem. Notice how the hydroxyl groups of 40 and 41 are forming H-bonds locking in those residues. Further, A1338 (A1996 in the eukaryotic ribosome) formed a *trans*-Hoogsteen/Watson–Crick pair (a “sheared” base pair) with G944 (G1521, respectively). Drawings made using PyMOL (PyMOL 1.7.7.6—Incentive Product © Schrodinger LLC).

The base pair between nucleotides 30 and 40 makes important contacts with the ribosome during translation (Fig. 3). In the bacterial P-site, the tRNA residues 30 and 40 contact A1339 of the 16S rRNA (A-minor type interaction), with G1338 forming an A-minor contact with the preceding pair 29–41 (generally a Watson–Crick pair) (Selmer et al. 2006). Similar contacts are formed between the 30–40 pair and A1996 as well as between the 29–41 pair and G1995 in eukaryotic ribosomes in *Leishmania* (Shalev-Benami et al. 2017) or involving A1576/G1575 in *Saccharomyces cerevisiae* (Tesina et al. 2020). In a GoU wobble, the U is displaced in the major groove of the helix compared to the C in a G = C pair. Thus, in a G30oU40 wobble pair, U40 would be displaced into the major groove, away from the minor groove, thereby preventing the formation of H-bonding contacts with A1339(A1996). Alternatively, a movement of G30 into the minor groove with the maintenance of the H-bonding contacts between U40 and A1339(A1996) would require movements of many residues because A1339(A1996) forms a pair with G944(G1521). Besides, residue U40 is modified into pseudouridine (Sprague et al. 1977) and this modification would enforce the wobbling, because a pseudouridine does not tautomerize (Westhof et al. 2019).

On the other hand, with the reversed U30oG40 pair, the contacts between A1339(A1996) and G40 can form (with N3 of G40 H-bonding to N2 of A1339), as in C30 = G40 (a combination observed in tRNA^Ala^ of fungi). Pairs involving A and U at positions 30 and 40 would allow similar contacts with A1339. In eukaryotes, the only tRNAs with a C30 = G40 pair are found also in tRNAs for His and Leu (CAG). In the CGC-tRNA^Ala^ of *B. mori*, the U29oU41 pair should not disrupt the contact with G1338(G1995).

#### Two base pairs in the tertiary structure are unusual in tRNA^Ala^

We first recall some observations about two structural pairs, essential for the maintenance of the tRNA L-shape fold, the non-Watson–Crick 15/48 and 54/58 pairs.

##### The 15–48 trans-Watson–Crick/Watson–Crick tertiary pair

Nucleotides at positions 15 and 48 form in tRNA structures a *trans*-Watson–Crick/Watson–Crick pair with, in most cases, position 15 a purine and position 48 a pyrimidine (Supplemental Fig. S1A). In bacteria and eukaryotes, both G15 = C48 and A15–U48 occur (see UCC-tRNA^Gly^ of *B. mori* in Fig. 1B). In archaea, only G15 = C48 pairs occur (with G15 modified into archaeosine, or 7-formamidino-7-deazaguanosine) (Watanabe et al. 1997). In *E. coli* tRNA^Cys^, a G15/G48 pair is present (Supplemental Fig. S1C; Hou et al. 1993). Such a G15/G48 pair is found in many other γ-proteobacteria (like *Enterobacter*, *Klebsiella*, *Salmonella*, or *Shigella*) but in neither vertebrates nor insects. Interestingly, in bacteria, there is a strong conservation of a G27oU43 wobble pair, whereas, in vertebrates and insects, that pair is reversed into U27oG43. There are other differential conservations between bacteria and eukaryotes. In bacteria, tRNA^Cys^ occurs mainly with A9, A13oA23 (or G9, G13oA22), and Y21, irrespectively of G15oG48 or G15 = C48. In eukaryotes, tRNA^Cys^ prefers A9, C13 = G22, and A21 always with C15 = G48. In both, there is a preference for Y60 and the possibility to form a pair between Y16 and residue 59 (that can be either R or Y).

##### The T-loop trans-Watson–Crick/Hoogsteen 54–58 tertiary pair

Nucleotides 54 and 58 in the T-loop stack on the last pair of the T-stem (a conserved G53 = C61) and form a *trans*-Watson–Crick/Hoogsteen pair between the highly conserved thymine at 54 and adenine at 58 (Supplemental Fig. S1B). The presence of the purine–purine A54oA58 pair is known in the T-loop of eukaryotic tRNA_i_^Met^ (Basavappa and Sigler 1991). A similar type of pair can be formed between the Watson–Crick edge of A54 and the Hoogsteen edge of A58, but it is longer than the T54oA58 pair (12.5 Å vs. 9.8 Å) (Supplemental Fig. S1D; Leontis et al. 2002). The residue A58 is modified in m1A in eukaryotic initiator tRNAs (Boccaletto et al. 2018) and the effects of A58 hypomodification investigated (Saikia et al. 2010). Two characteristic features of the initiator tRNA_i_^Met^ are three G = C pairs in the anticodon stem (Gs on the 5′ strand and Cs on the 3′ strand) before the anticodon loop and an A54oA58 pair in the T-loop.

##### The R15oA48 and the T-loop A54oA58 pairs occur together in AGC-tRNA^Ala^

In tRNA^Ala^, the trends are different, especially for the A(I)GC anticodons. In bacteria and archaea, there is no tRNA^Ala^ starting with A34 (Grosjean et al. 2010). However, A(I)34-containing tRNA^Ala^ do occur in eukaryotes. We did not observe these pairs in fungi or nematodes. Surprisingly, in such A(I)GC anticodons of other eukaryotes, the two purine–purine pairs, 15–48 and 54–58, occur simultaneously in a large proportion of sequences analyzed (with or without G30oU40). Residue R15 is mainly G15, but A15 does occur depending on the phyla. The residues R15, A48, and A54 are not modified, but the residue 58 is m1A58 (Fig. 2A,C).

**FIGURE 4.**
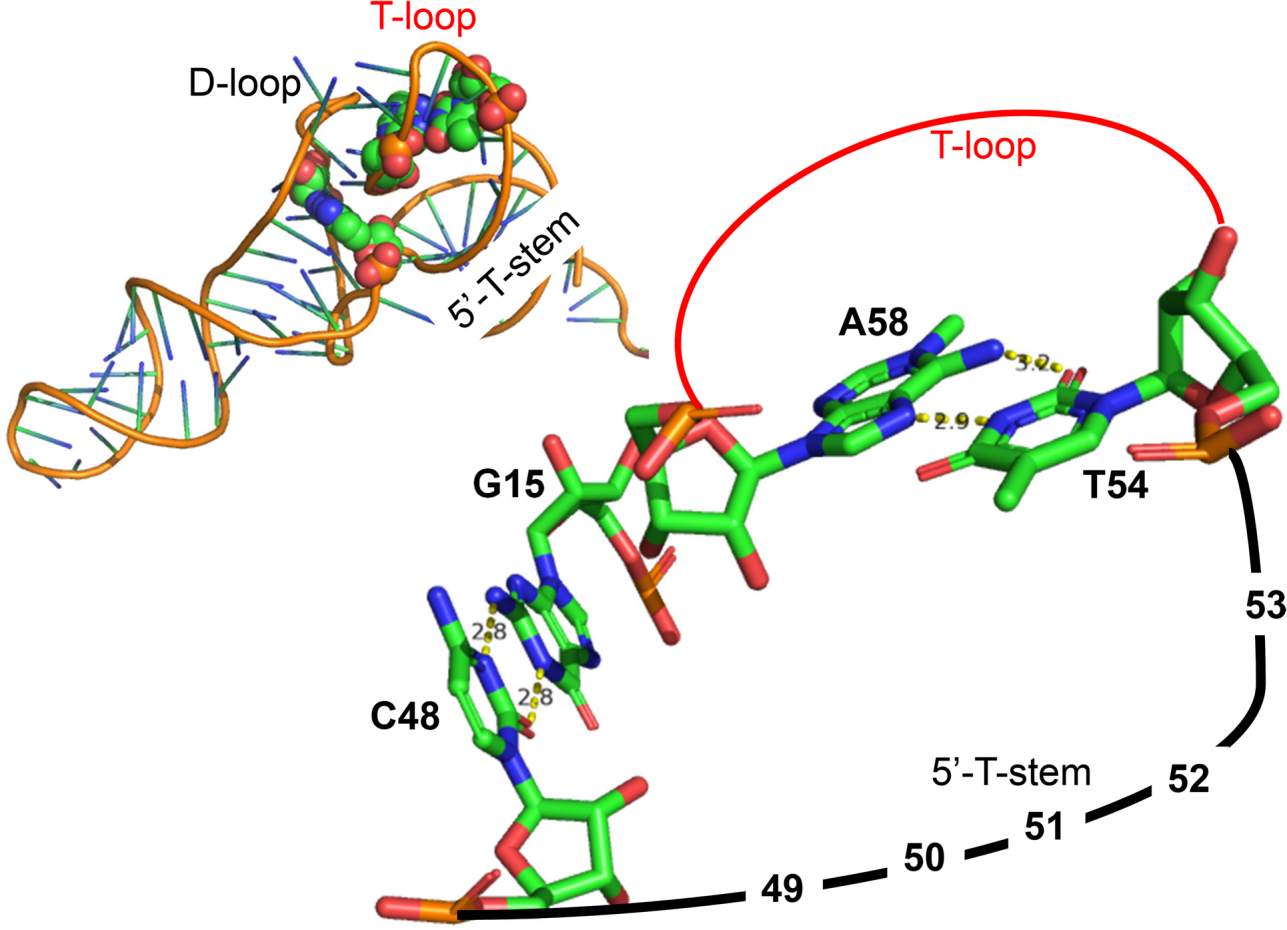
On the *top left*, a tRNA structure (PDB 1EHZ from Shi and Moore 2000) is shown with the two non-Watson–Crick pairs 15/48 (linking the beginning of the D-loop to the end of the variable loop) and 54/58 (closing the T-loop) highlighted with van der Waals spheres. On the *right*, a simplified diagram shows that both 48 and 54 are on the same strand so that a change from a pyrimidine to a purine would draw toward the reader the strand (with probably accompanying movement of the T-helix). Drawings made using PyMOL (PyMOL 1.7.7.6—Incentive Product © Schrodinger LLC).

These unusual pairs occur together with an additional residue in the D-loop, U17 (modified in dihydrouridine [D] D17), and the presence of a purine at positions 16 and 60 in the T-loop. In T-loops, a purine at 59 is more frequent than at position 60 (for steric constraints). In some crystal structures—for example, in tRNA^Cys^ (Hauenstein et al. 2004)—residue Y16 has been observed paired *trans*-Watson–Crick/Watson–Crick with Y59. In tRNA^Ala^, R16 could still form a *trans*-pair with Y59 and R60 stacking over. In which case, the additional U17(D17) residue would bulge out of the loop. Purine–purine pairs are longer than purine–pyrimidine pairs, and the way such pairs are accommodated in that key region of the tRNA core will depend on the immediate environment. Figure 4 illustrates the relative orientations and relationships between residues 48 and 54: If one moves in a direction, the other one should follow. The pair 54–58 has already been observed and discussed (see above). But what is the nature of the 15–48 bp? It can be either A15/A48 or G15/A48. Among non-Watson–Crick pairs, isosteric A/A and G/A pairs exist in the *trans*-Sugar-Edge/Hoogsteen family (as in GNRA tetraloops). But either a single H-bonded pair or a bifurcated pair (Supplemental Fig. S1C), like the one between G15/G48 in tRNA^Cys^ (Nissen et al. 2009), is probable.

## DISCUSSION

**FIGURE 5.**
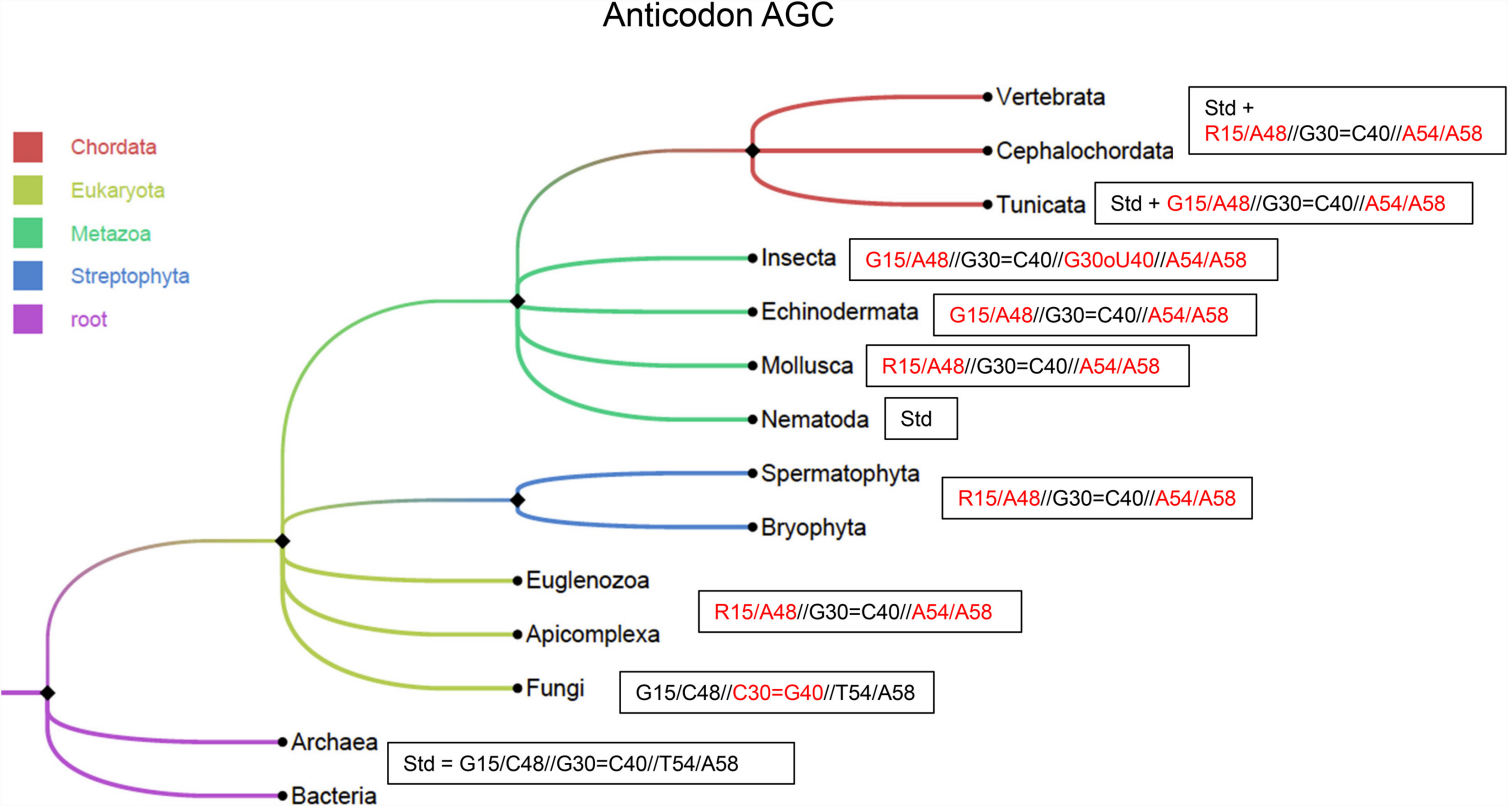
Presence of unusual pairs in AGC-tRNA^Ala^ isodecoders in various branches of the phylogenetic tree. We checked the tRNA predictions present in GtRNAdb (Chan and Lowe 2016). As there were too many sequences, we used a threshold for the tRNAscan-SE score of 60. For the Spermatophyta, this represents 242 sequences and for the Vertebrata 199. The proportions of AGC-tRNA^Ala^ isodecoders vary strongly depending on species. Standard base pairs are in black and unusual pairs in red. The tree was built by the Taxonomy Common Tree tool (https://www.ncbi.nlm.nih.gov/Taxonomy/CommonTree/wwwcmt.cgi) and drawn using the software FigTree v1.4.4 (https://github.com/rambaut/figtree/).

Several of these observations can be found scattered in the literature (Garel and Keith 1977; Sprague et al. 1977; Zuniga and Steitz 1977; Kawakami et al 1978; Fournier 1979). What the present comparisons (see Table 1) show is that (i) eukaryotic AGC-tRNA^Ala^ present unusual tertiary pairs: a GoU instead of a G = C pair in the anticodon, a pair recognized in the P-state, and two pairs that occur together, R15/A48 and A54/A58; (ii) in mammals, the YGC anticodons use the G30oU40 pair and the AGC ones the combination of both A15/A48 and A54/A58; (iii) in some insects (*Drosophila* species, *Bombyx* species, *Anopheles gambiae*), the AGC-tRNA^Ala^ present all unusual pairs, G30oU40 (G30oΨ40 in *B. mori*) and G15/A48 with A54/A58; in the *Bombyx* species, some AGC-tRNA^Ala^ present the G30 = C40 pair together with both G15/A48 and A54/A58; and (iv) the two purine–purine pairs occur with an additional residue in the D-loop and a purine at position 60 (instead of the usual pyrimidine). In Figure 5, the main trends are represented depending on some large divisions.

Emphasis was made here on non-Watson–Crick pairs and especially GoU wobble pairs because such pairs have major structural impact on the three-dimensional fold of RNA molecules. In non-Watson–Crick pairs, various edges are used for H-bonding positioning the sugar-phosphate backbone so that the strands can be parallel or the anionic phosphate oxygens turned inside the fold and not to its exterior (Leontis et al. 2002). The GoU wobble pairs induce an over- or undertwisting in a helical stem depending on their 5′ or 3′ position so that a GoU pair can position differently in an apical loop (Masquida and Westhof 2000). However, the functional implications of those unusual features in AGC-tRNA^Ala^ are difficult to nail down on the basis of sequence alone. These features could participate in the recognition processes with the aminoacyl tRNA synthetases, with ribosomal states, or with both, and structures of complexes between such tRNAs and aminoacyl tRNA synthetases or the ribosomes would be required. The known structure of a complex between tRNA^Ala^ and aminoacyl tRNA synthetase is with the aminoacyl tRNA synthetase from the archaeon *Archaeoglobus fulgidus* (Naganuma et al. 2014). In that complex, the synthetase, besides contacting tightly the conserved G3oU70 pair, contacts also residues 12, 13, 20, and 47 and the conserved G19 = C56 pair at the extremity of the T-loop. Comparisons between yeast tRNA^Asp^ free and in complex with its cognate aminoacyl synthetase show that deviations between the two tRNAs occur at a hinge point formed by the yeast-specific G30oU40 pair in the AC-stem (Ruff et al. 1991). In addition, residues 15, 54, and 58 involved in some of the unusual pairs belong to the A- and B-boxes for transcription by polymerase III (Marck et al. 2006; Mitra et al. 2015).

The presence of a G30oΨ40 wobble pair at a recognition point of the tRNA anticodon stem in the P-state could imply a role of that pair during translocation. It is known from past literature that, of the two isoacceptor tRNA^Ala^ species in *B. mori*, the one with the G30oΨ40 pair is twice as highly expressed in the posterior glands of the silkworm where silk translation occurs (Garel et al. 1974; Meza et al. 1977; Sprague et al. 1977). Interestingly, a similar phenomenon (Garel et al. 1974; Garel and Keith 1977; Zuniga and Steitz 1977) occurs for tRNA^Gly^, where the most expressed isoacceptor in the posterior silk gland has the anticodon GCC in which there is a U28oG42 pair preceding the C29 = G41 pair. The C29 = G41 pair is recognized during translation in the P-state by an A-minor type contact with a G (Fig. 3; Selmer et al. 2006; Shalev-Benami et al. 2017). The presence of a 5′ U28oG42 pair will lead to a strong unstacked conformation (Masquida and Westhof 2000) with the following C29 = G41 so that G41 will lose its contact to the purine residue in the rRNA.

The two tRNA species discussed here (Ala and Gly) are major components of silk fibroin (∼30% and ∼43%, respectively [Fournier 1979]) and both display different peculiarities. For example, the aminoacyl tRNA synthetase specific for Ala does not recognize the anticodon of tRNA^Ala^ (Naganuma et al. 2014) and, in Eukarya, tRNA^Gly^ is the only tRNA species related to four-codon boxes with a G34 and not a A34(I) at the first base of the anticodon triplet (Grosjean et al. 2010). Also, both tRNA species lead to a high-GC content of the codon/anticodon triplet helix and require special conservations in the anticodon loop to guarantee smooth and uniform decoding in bacteria, especially at positions 32 and 38 (Ledoux et al. 2009; Murakami et al. 2009; Grosjean and Westhof 2016; Pernod et al. 2020). One can therefore wonder whether the observed GoU pairs in the anticodon helix of eukaryotic tRNA^Ala^ and tRNA^Gly^ do not contribute to smooth and uniform decoding.

## MATERIALS AND METHODS

The analysis is based on the database of transfer RNA genes GtRNAdb 2.0 (Chan and Lowe 2016). The database contains alignments of tRNA genes based on the tRNAscan-SE prediction algorithm (Lowe and Eddy 1997). The sequences are organized as a function of an overall bit score. The score is composed of a primary sequence score and a secondary structure score based on the covariance model. A score below 55.0 may indicate the presence of a pseudogene. There are several isodecoders for the isoacceptor tRNAs (Goodenbour and Pan 2006). But, for most genomes, only a fraction of the predicted isodecoder tRNA genes have generally been experimentally observed and the tRNA modifications are known for still a smaller fraction of those on the basis of the MODOMICS database (Boccaletto et al. 2018). We extracted the tRNA alignment from the GtRNAdb 2.0 and realigned structurally by taking care of known tertiary structure conservations.

**TABLE 1.**
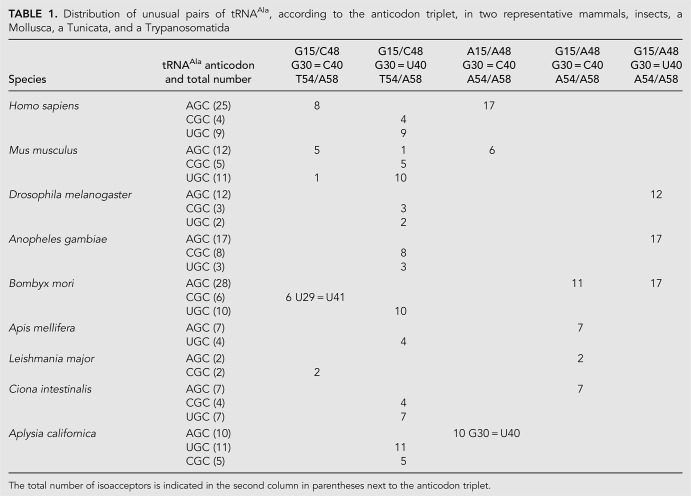
Distribution of unusual pairs of tRNA^Ala^, according to the anticodon triplet, in two representative mammals, insects, a Mollusca, a Tunicata, and a Trypanosomatida

## SUPPLEMENTAL MATERIAL

Supplemental material is available for this article.

## Supplementary Material

Supplemental Material
