# Supplementary material for: Unusual tertiary pairs in eukaryotic tRNAAla
Source: RNA. 2020 Nov;26(11):1519–29. doi: 10.1261/rna.076299.120 (PMC7566577; doi:10.1261/rna.076299.120)
Supplement: Supplemental Material [file supp_26_11_1519__index.html]

Unusual tertiary pairs in eukaryotic tRNA-Ala — Unusual tertiary pairs in eukaryotic tRNAAla — Supplemental Material 

# Unusual tertiary pairs in eukaryotic tRNAAla

## Supplemental Material

- Supplemental\_Material.docx
