# Supplementary material for: Unusual tertiary pairs in eukaryotic tRNAAla
Source: RNA. 2020 Nov;26(11):1519–29. doi: 10.1261/rna.076299.120 (PMC7566577; doi:10.1261/rna.076299.120)
Supplement: Supplemental Material [file supp_076299.120_Supplemental_Material.docx]

**Unusual tertiary pairs in eukaryotic tRNA^Ala^**

Eric Westhof ^1,2*^, Shubo Liang^1^, Xiaoling Tong^1^, Xin Ding^1^, Lu Zheng^1^, Fangyin Dai^1^

^1^State Key Laboratory of Silkworm Genome Biology; College of Biotechnology, Southwest University, Chongqing 400715, China.

^2^Architecture et Réactivité de l’ARN, Institut de Biologie Moléculaire et Cellulaire, UPR9002 CNRS, Université de Strasbourg, Strasbourg 67084, France.

Shubo Liang : shuboliang@email.swu.edu.cn

Xiaoling Tong : xltong@swu.edu.cn

Xin Ding : dingx305@163.com

Lu Zheng : zhengluluxi@163.com

Fangyin Dai : fydai@swu.edu.cn

*** Correspondence:** Eric Westhof : e.westhof@ unistra.fr

Keywords: tRNA, anticodons, Ala, Gly, *B. mori*, insects, mammals.

**Supplementary material**

**Figure S1:** Drawings of tertiary pairs discussed in the text. Top left (A): the *trans* Watson-Crick/Watson-Crick G15/C48 (from PDB 1EHZ). Top right (B): the *trans* Watson-Crick/Hoogsteen T54/m1A58 (from PDB 1EHZ). Bottom left (C): the bifurcated pair between G15 and G48 in tRNACys (from PDB 2DU3). Bottom right (D): the *trans* Watson-Crick/Hoogsteen A54/m1A58 (from (1YFG). Drawings made using Pymol (PyMOL(TM) 1.7.7.6 – Incentive Product Copyright © Schrodinger LLC.

**
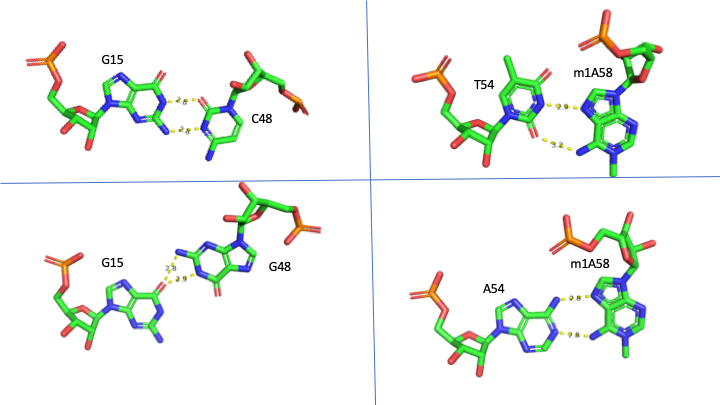
**
